# Association between Poor Ergophthalmologic Practices and Computer Vision Syndrome among University Administrative Staff in Ghana

**DOI:** 10.1155/2020/7516357

**Published:** 2020-04-27

**Authors:** Samuel Bert Boadi-Kusi, Sampson Listowell Abu, George Oppong Acheampong, Peter Osei-Wusu Adueming, Emmanuel Kwasi Abu

**Affiliations:** ^1^Department of Optometry and Vision Sceinces, School of Allied Health Sciences, College of Health and Allied Sciences, University of Cape Coast, PMB, Cape Coast, Ghana; ^2^Department of Ophthalmology and Visual Sciences, School of Medicine, University of Alabama at Birmingham, Birmingham, AL, USA; ^3^Eye Unit, DelCielo Optical Services, P. O. Box WY 2244, Kwabenya, Accra, Ghana; ^4^Laser and Fibre Optics Centre, Department of Physics, School of Physical Sciences, College of Agriculture and Natural Sciences, University of Cape Coast., Cape Coast, Ghana

## Abstract

**Aim:**

The aim of this study was to assess the prevalence of computer vision syndrome (CVS) and its associated ergonomic factors among university administrative staff in Ghana.

**Methods:**

A cross-sectional survey was conducted among 200 administrative staff of the University of Cape Coast. The procedure included a self-administered questionnaire, comprehensive ocular health examination, and assessment of computer workstation and lighting conditions. The prevalence of CVS among the subjects and the association between CVS and ergonomic practices were determined.

**Results:**

The mean age of the study sample was 31.0 ± 4.7 years, and the majority were males (56.0%). The prevalence of CVS was among 103 (51.5%)participants. Over a third of the respondents used computers for 6 or more hours daily. Significant association was found between CVS and poor ergonomic practices (*χ* = 15.175, *p* = 0.001).

**Conclusion:**

In addition to poor ergonomic office setup, university administrative staff spend several hours behind computer screens leading to the development of CVS. Increased awareness of CVS and adherence to recommended ergonomic practices are necessary to reduce the prevalence of CVS and ultimately enhance work satisfaction and productivity.

## 1. Introduction

The advent of computers in the 20th century is arguably the biggest technological revolution following the industrial revolution. Owing to their high efficiency and varied applications in the 21st century, the use of computers has proliferated and become ineluctable at recreation facilities, homes, and workplaces such as the academic institutions [1, 2]. The extensive use of computers, particularly at workplaces, has unfortunately compounded work-related health complaints and symptoms such as ocular problems, musculoskeletal discomfort, and stress [3, 4]. Vision-related problems are the most frequently reported health-related problems occurring in majority of computer users [3, 5, 6]. Prolonged daily use of computers has been identified to be a major precursor to developing computer vision syndrome (henceforth CVS) [6–9].

The American Optometric Association aptly defines CVS as a complex of eye and vision problems related to near vision tasks which occurs during and/or after the use of computers and prolonged viewing of the video display terminals (henceforth VDT) [10]. Common symptoms of CVS include but not limited to the following: dry and irritated eyes, excessive tearing, eye strain, hyperemia, burning sensation, blurred vision, diplopia, headache, glare sensitivity, transient poor color perception, and neck or shoulder pain [1, 2, 10–12]. These symptoms are noted to arise when the visual demands while working on a VDT exceed the abilities of the user [13]. Approximately 70% of computer users suffer from CVS [3]. Izquierdo et al. reported that the global prevalence of CVS ranges from 25% to 93% [14].

The cause of visual problems experienced while using computers is multifactorial. While there is no strong evidence for causation [15], previous studies have reported that visual symptoms increased with the prolonged exposure to computer screens [16–18]. Uncorrected refractive error, presbyopia, and binocular vision abnormalities are additional factors associated with computer-related visual symptoms [6, 10]. For instance, the incidence of CVS has been reported to be higher in both undercorrected and uncorrected ametropes than in emmetropes [19, 20]. Wiggins and Peers showed that uncorrected astigmatism produced a significant increase in CVS symptoms [21]. Environmental factors, poor computer design, and workplace ergonomics also contributed to the development of symptoms and complaints of CVS. In addition, poor lighting, imbalance between light of the computer screen and the surrounding, age, gender, and systemic diseases have also been enumerated by Rosenfield as environmental or external factors that risk the development of CVS [12].

Workplace ergonomics refers to the arrangement of equipment and furniture in an office space for users to work more efficiently and comfortably [22]. Visual or computer ergonomics on the other hand involves proper positioning of keyboard, monitor, mouse, chairs, desks, document folders, seat height, seat width and depth, seat material, backrest, materials in the office, armrests, leg room, thickness of work surface, footrest, document holder, wrist rests, and so on [15, 23, 24]. In a Nepalese study, users with their computer screens set below the eye level had significantly lower CVS case than those who viewed the screen at or above the eye level [25]. Mashige and colleagues studied ergonomic factors associated with CVS among nonpresbyopic university staff in South Africa [26]. They found that, in addition to poor ergonomic set up at workstations, participants were ignorant of ergonomic standards for computer use. Many researchers have prescribed measures to alleviate the symptoms of CVS. These include frequent blinking (12–18/minute), short-time breaks after every 20 minutes to look at distant object at least 20 feet away for 20 seconds (20–20–20 rule), adjustment of workstation, good sitting posture, appropriate lighting and screen brightness, and regular stretching of arms, leg, back, neck, and shoulders [10, 23, 27].

University administrative staff find themselves among the cohort of people who are more predisposed to developing CVS as they spend several hours daily behind computer screens. As office duties become more computer dependent, CVS will remain a significant health problem with its socioeconomic ramifications ranging from low productivity to poor job satisfaction. The aim of the present study was to determine the prevalence of CVS and identify the associated factors among university administrative staff in Ghana.

## 2. Materials and Methods

### 2.1. Study Population and Sampling

A cross-sectional study was carried out among the administrative staff of the University of Cape Coast in Ghana. Following study approval by the Institutional Review Board, 308 University Administrative staff were enumerated based on a systematic selective sampling technique using data obtained from the Human Resource Division of the University of Cape Coast. We recruited administrators, who in the preceding 6 months, averaged a daily minimum of 5 screen hours and had no systemic disease, binocular vision anomalies, or other ocular health conditions. Excluded participants were those aged 40 years and above, early presbyopes, refractive error above ±3.75 D, corrected visual acuity worse than 6/6, pregnancy, use of oral contraceptives, migraine, contact lens wear, and use of short-term or long-term systemic medication.

### 2.2. Data Collection

Each subject signed a consent form before participating in the study. The data collection required the subjects to undergo three procedures administered by three trained personnel. First, each participant answered an adopted and validated computer vision syndrome questionnaire (CVS-Q) [28]. The questionnaire probed into demographics, CVS symptoms, computer-contact hours, breaks, and their ergonomic factors that might affect a respondent's comfort while using the computer. Study participants then underwent a comprehensive ocular health examination. This examination spanned from case history, visual acuity, ophthalmoscopy, refraction, and binocular vision assessment. This was done to ensure that participants satisfied the inclusion criteria set for the study. Based on the presented complaints, that is, the questionnaire responses, any participant who scored ≥6 points, as indicated by the CVS-Q [28], was assigned to either a CVS case category or non-CVS category.

The third phase involved observation and measurement of ergonomic parameters at each administrator's workstation. The lighting around the computer workstation was measured with a calibrated digital light meter (Model 403125, Extech Instruments, USA). The following parameters (with their expected values) were recorded at each workstation:

- The viewing angle of the participant's eye level to the top of the computer screen (10°–20°)
- The viewing angle of the participant's eye level to the center of the computer screen (21°–30°)
- The viewing angle of the participant's eye level to the position of bottom of the screen (31°–40°)
- The viewing distance from the horizontal to the top of the screen (18–28 cm)
- The viewing distance from the horizontal to the bottom of the screen (40–60 cm)
- The viewing distance from the eye to horizontal center of the screen (50–70 cm)
- Viewing distance from the eye to the keyboard (63–82 cm)
- Height of the keyboard from the floor (60–82 cm)
- Light intensity between participant and computer (75–150 Cd/m^2^)
- Light intensity of room (200–500 Cd/m^2^)

These expected values were adapted from the ergonomic recommendations by the Workers' Compensation Board (WCB) of British Columbia [29], Oregon Occupational Safety and Health Agency [30], and ANSI/HFES 100-2007 [31]. Applicable viewing angles were computed in degrees using the formula: Tan *α* *=* *c*/*b*, where *α* equals the viewing angle, *b* is the horizontal viewing distance from computer screen to participant, and *c* is the viewing distance from top of computer screen to eye level. Any participant who recorded more than three measurements out of the ten parameters assessed, falling outside the recommended range, was classified under “poor ergonomic practice.”

**Figure 1:**
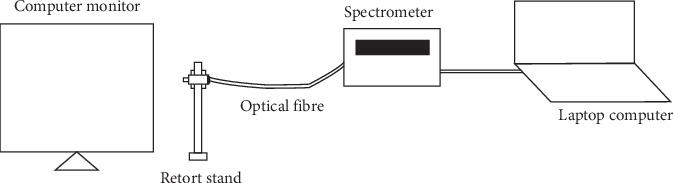
Schematic diagram for measuring light radiation from computer monitor.

Finally, the spectra of radiations from some sampled computer monitor screens used by the administrative staff were measured in an experimental setup as schematically shown in Figure 1. The setup consisted of an optical fibre (BIF600-UV-VIS, Ocean Optics), a spectrometer from ocean optics (USB 4000 Spectrometer, Ocean Optics), and a laptop computer (Toshiba Laptop (3.0 GHz 8.0 GB, AMD A10-4600M).

In acquiring the spectra of radiations from the sampled computer monitors, radiations were coupled into the optical fibre to the spectrometer which is connected to the laptop computer. The spectra from the radiations were then displayed on the laptop computer with the aid of spectra suite software. The spectra data were then extracted for further analysis.

### 2.3. Data Analysis

All data were entered and analyzed using Statistical Package for Social Sciences (SPSS version 21.0.). Seven out of ten measured workstation parameters within the expected range of values were deemed “good ergonomic practice,” otherwise classified as “poor ergonomic practice.” Using the Likert scale, frequency of CVS symptoms was rated from Never to Often or Always while intensity was rated from Moderate to Intense or Severe [28]. Descriptive statistics was used to explore the association between ergonomic factors and computer vision syndrome with the level of significance set at 0.05.

## 3. Results

### 3.1. Participant Characteristics

Out of the 308 invitees, 200 subjects enrolled and completed the study (response rate—65.0%). The remaining 108 either declined to participate in the study or could not complete the study due to busy work schedule. The mean age of the respondents was 31.0 ± 4.7 years (range 19–39 years) and 56.0% of the 200 respondents were males. A majority (76.0%) of the study population fell within the age category of 25–35 years. Almost half of the study population had worked with computers for more than 5 years. Fifty-nine percent of the respondents took a break from their computers whenever they felt tired. Of the 200 participants, 103 (51.5%) were found to have symptoms of CVS. Nine percent more males than females had CVS. Sample characteristics are summarized in Table 1. More than one-third (37.5%) of the participants aged 25–29 years were exposed to computer screens for at least 6 hours daily. The number of hours spent daily on using computers by participants in each age category are represented in Table 2.

### 3.2. Frequency and Severity of CVS Symptoms

The most frequent moderate to severe symptoms were burning sensation, foreign body sensation, eye pains, itching, and blurred vision. The distribution of the severity of CVS symptoms is shown in Table 3.

### 3.3. Ergonomic Measures and Association with the Prevalence of CVS

The ergonomic measurements, the expected values, and their association with good or poor practices are presented in Table 4. There was a significant association between CVS case category and wrong viewing angle to the center of the computer screen in 73.5% of the respondents. The office illumination and light intensities emitted from the computers were found to be suboptimal in 99.0% of the workstations of those belonging to the CVS case group. Overall, poor ergonomic practices were observed at the workstations of 79.5% of the study population. We found a significant association between CVS and poor ergonomic practices (*X* = 15.175, *p*=0.001) as indicated in Table 5.

**Figure 2:**
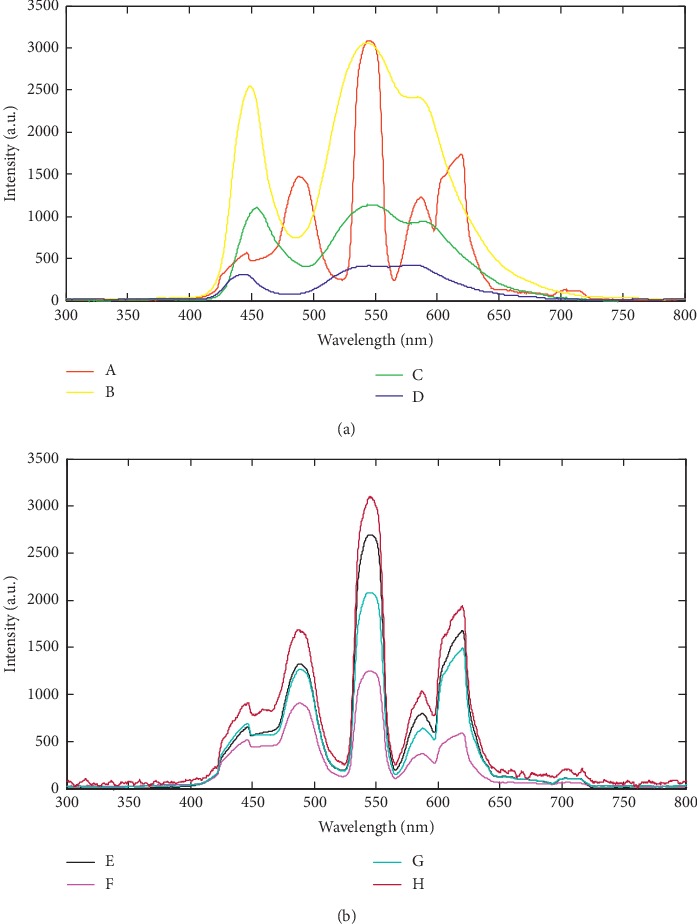
(a) Spectra of light radiation from different sampled computer screens. (b) Spectra of light radiation from different sampled computer screens.

Figures 2(a) and 2(b) illustrate the distribution of the measured light intensities from the computer monitor screens at some observed workstations. It is evident from the figures that blue light was being emitted from some of the sampled computer screens.

## 4. Discussion

The present study investigated the prevalence of computer visual syndrome and its associated risk factors among university administrative staff. This study population was chosen because they are part of a group of professionals who are at a greater risk of developing computer-related visual problems. The University of Cape Coast operates a decentralized administrative system that employs more administrative personnel among whom we recruited a sample for the study. Our study sample was comprised of more males than females and a mean age of 31.0 ± 4.7 years. Related studies done on CVS in Ghana [32], Nigeria [33], and South Africa [26] had similar age and gender distribution, except for the South African study [26] that had more females than males. The prevalence of CVS signs and/or symptoms was found to be 51.5% and the presence of CVS was associated with poor ergonomic practices or workstation setups.

In contrast to our study, a higher prevalence of CVS has been reported for different cohorts of computer users elsewhere: 67.4% in Sri Lanka [8], 74.0% in Nigeria [33], and 80.3% in Chennai, India [34]. In the Sri Lankan study, Ranasinghe et al. followed 2210 computer users of two telecommunication institutes in nine provinces for a year. The yearlong follow-up with significantly larger sample size and inclusion of older subjects (age range:18–60 years) may explain the higher prevalence of CVS cases in the Sri Lankan study [8] compared to our study. Again, in that study, a higher prevalence of CVS (69.5%) was reported for females in a male dominated study sample contrasting our finding of higher CVS cases (54.4%) in males. The higher prevalence of CVS among males in our present work may be a direct influence of the imbalanced gender distribution of study participants.

We also observed that approximately 50% of the CVS cases were within the age bracket of 25–29 years. This finding is corroborated by the fact that young individuals tend to use computers for long hours and the same reason may hold for the higher prevalence of CVS reported for medical and engineering students in Chennai, India [34]. It is important to mention that in addition to including the vulnerable contact lens wearers in study sample, the presence of neck and shoulder pains counted towards the prevalence of CVS symptoms in the Chennai study. The presence of presbyopia [9, 10], uncorrected ametropia [10, 20], and contact lens wear [21] may exacerbate visual symptoms frequently reported by CVS patients. To ensure that the reported symptoms were as a result of computer usage and consistent with Mashige et al. [26], persons with the following attributes were excluded from our study sample: early presbyopes, individuals 40 years and above, greater than ±3.75 D refractive error, contact lens wear, systemic and ocular diseases, pregnancy, and use of oral contraceptives.

While headaches and increased sensitivity to light were identified as the most frequent severe CVS symptoms, burning sensation, itching, and eye pain were the most frequent moderate symptoms enumerated by the participants. Rosenfield posits that burning sensation and irritation are due to dryness of the ocular surface caused by infrequent blinking of the eyes during the use of computers [10]. Majority (53.0%) of the participants reported more than 5 hours of daily screen time without taking regular breaks. Infrequent breaks and prolonged use of computers are strongly associated with complaints of dryness of the ocular surface. We also found similar symptoms identified by Sheedy [19] as the five commonest CVS symptoms: eyestrain, headache, blurred vision, dry eyes, and neck/back pain.

Poor workstation or office ergonomic practices account for several visual symptoms and all non-eye-related symptoms associated with the use of computers [22]. Inappropriate positioning of the computer and its accessories and improper viewing angles cause aching of the muscles of the neck, shoulder, and back [4, 22, 23]. Severe musculoskeletal complaints were also reported by 63 out of the 103 participants, classified as having CVS, and 45 out of the 97 respondents without CVS. Furthermore, there was a significant association between the presence of CVS and poor ergonomic practices (*χ* = 15.175, *p* = 0.001). Assessment of the participants' workstations revealed wrong viewing angle to the center of the screen, improper viewing distance, wrong positioning of keyboard, suboptimal office illumination, and improper computer screen brightness. CVS symptoms such as glare and asthenopia are also associated with improper screen brightness, wrong viewing angle and distance, and poor room illumination [6, 26]. These improper ergonomic practices were more prevalent among the CVS case group. Ranasinghe and colleagues showed that users at workstations which were noncompliant to standard ergonomic recommendations had higher prevalence of CVS [8]. It is important to note that some of the computer screens sampled emitted blue light which has been documented to be injurious to the unprotected eyes [35–37]. Protection of eyes with antireflective lenses or computer screen shields may be helpful. There is, however, no strong evidence backing advantages of using blue light blocking lenses or computer screen shields [38].

Of note, we did not test the association between participants' awareness and the presence of CVS because we did not obtain participants' knowledge on CVS and office ergonomics. Contrary to other studies [25, 34], we did not categorize musculoskeletal pain as a symptom of CVS. Since we were interested in the association between workstation ergonomics and CVS, we did not assess participants' anthropometry information. Even though there is no gold standard to diagnose CVS, we navigated this shortcoming by adopting previously used definitions and criterion to study CVS. It is anticipated that subsequent follow-up studies would take into considerations these limitations.

## 5. Conclusion

There is a high prevalence of computer vision syndrome among university administrative staff. Younger employees had prolonged daily use of computers, increasing their chances of developing CVS. Incorrect viewing angle and distance as well as poor office lighting are among the many ergonomics factors associated with CVS among computer users. Further studies in larger sample will help to better understand the magnitude of the burden CVS poses to the health system and national economy. As access to computer continues to become universal at many workplaces, increasing public awareness and compliance to recommended office ergonomics are necessary measures to mitigate the rising prevalence in CVS cases.

## Figures and Tables

**Table 1:** Distribution of parameters of administrators according to cases and noncases.

Distributions		CVS status		Total (*n*, %)
		Cases (*n*, %)	Noncases (*n*, %)	
Age range	<0.25	2 (1.9)	3 (3.1)	5 (2.5)
	25–29	46 (44.7)	39 (40.2)	85 (42.5)
	30–35	36 (35.0)	31 (32.0)	67 (33.5)
	36–40	19 (18.4)	24 (24.7)	43 (21.5)
	Total	103 (100)	97 (100)	200 (100)

Gender	Male	56 (54.4)	56 (57.7)	112 (56.0)
	Female	47 (45.6)	41 (42.3)	88 (44.0)
	Total	103 (100)	97 (100)	200 (100)

Years of working with computer	2–5	52 (50.5)	46 (47.4)	98 (49.0)
	6–9	27 (26.2)	23 (23.7)	50 (25.0)
	10–14	14 (13.6)	22 (22.7)	36 (18.0)
	15–20	10 (9.7)	6 (6.2)	16 (8.0)
	Total	103 (100)	97 (100)	200 (100)

Educational background	SHS	2 (1.9)	1 (1.0)	3 (1.5)
	Diploma	6 (5.8)	8 (8.2)	14 (7.0)
	First degree	73 (70.9)	63 (64.9)	136 (68.0)
	Second degree	22 (21.4)	25 (25.8)	47 (23.5)
	Total	103 (100)	97 (100)	200 (100)

Musculoskeletal symptoms	Never	7 (6.8)	16 (16.5)	23 (11.5)
	Mild	11 (10.7)	14 (14.4)	25 (12.5)
	Moderate	22 (21.4)	22 (22.7)	44 (22.0)
	Severe	63 (61.2)	45 (46.4)	108 (54.0)
	Total	103 (100)	97 (100)	200 (100)

Computer breaks	No break	3 (2.9)	9 (9.3)	12 (6)
	Every 20 min	30 (29.1)	14 (14.4)	44 (22)
	Every hour	18 (17.5)	8 (8.2)	26 (13)
	When tired	52 (50.5)	66 (68)	118 (59)
	Total	103 (100)	97 (100)	200 (100)

**Table 2:** Age groups and hours spent on computer per day.

Age group	2–5 hours (%)	6–9 hours (%)
<25	1 (0.5)	4 (2.0)
25–29	10 (5.0)	75 (37.5)
30–35	47 (23.5)	20 (10.0)
36–40	36 (18.0)	7 (3.5)

**Table 3:** Severity of CVS symptoms among administrators.

CVS indicator	Never	Moderate	Severe	Total
Burning sensation	26 (25.2)	67 (65)	10 (9.7)	103 (100)
Itching	26 (25.2)	64 (62.1)	13 (12.6)	103 (100)
Foreign body sensation	48 (46.6)	53 (51.5)	2 (1.9)	103 (100)
Tearing	45 (43.7)	49 (47.6)	9 (8.79)	103 (100)
Excessive blinking	42 (40.8)	52 (50.5)	9 (8.7)	103 (100)
Eye redness	52 (50.5)	45 (43.7)	6 (5.8)	103 (100)
Eye pain	38 (36.9)	55 (53.4)	10 (9.7)	103 (100)
Heavy eyelids	58 (56.3)	33 (32)	12 (11.7)	103 (100)
Dryness	67 (65)	33 (32)	3 (2.9)	103 (100)
Blurred vision	41 (39.8)	50 (48.5)	12 (11.7)	103 (100)
Double vision	72 (69.9)	27 (26.2)	4 (3.9)	103 (100)
Difficulty focusing for near	71 (68.9)	27 (26.2)	5 (4.9)	103 (100)
Increased sensitivity to light	36 (35)	43 (41.7)	24 (23.3)	103 (100)
Circle of light around an object	80 (77.7)	18 (17.5)	5 (4.9)	103 (100)
Feeling that sight is worsening	67 (65)	35 (34)	1 (1)	103 (100)
Headache	27 (26.2)	53 (51.5)	23 (22.3)	103 (100)

**Table 4:** Ergonomic measurements of administrators.

Ergonomic parameters	Ergonomic practice	CVS case	Non-CVS case	Total	*X*	*p* value
Viewing angle from eye to top of computer screen	Poor	70 (68.0)	51 (52.6)	121 (60.5)	4.947	0.026
	Good	33 (32.0)	46 (47.4)	79 (39.5)		
	Total	103 (10)	97 (100)	200 (100)		

Viewing angle from eye to center of computer screen	Poor	87 (84.5)	60 (61.9)	147 (73.5)	13.112	0.001
	Good	16 (15.5)	37 (38.1)	53 (26.5)		
	Total	103 (100)	97 (100)	200 (100)		

Viewing angle from eye to bottom of computer screen	Poor	51 (49.5)	40 (41.2)	91 (45.5)	1.380	0.240
	Good	52 (50.5)	57 (58.8)	109 (54.5)		
	Total	103 (100)	97 (100)	200 (100)		

Distance from horizontal to top of computer screen	Poor	103 (100	68 (70.1)	171 (85.5)	36.016	0.001
	Good	0 (0.0)	29 (29.9)	29 (14.5)		
	Total	103 (100)	97 (100)	200 (100)		

Distance from horizontal to bottom of computer	Poor	74 (71.8)	56 (57.7)	130 (65.0)	4.373	0.037
	Good	29 (28.2)	41 (42.3)	70 (70.5)		
	Total	103 (100)	97 (100)	200 (100)		

Distance from horizontal to center of computer screen	Poor	71 (68.9)	48 (49.5)	119 (59.5)	7.840	0.005
	Good	32 (31.1)	49 (50.5)	81 (40.5)		
	Total	103 (100	97 (100)	200 (100)		

Viewing distance from eye to home row of keyboard	Poor	79 (76.7)	58 (59.8)	137 (68.5)	6.616	0.010
	Good	24 (23.3)	39 (40.2)	63 (31.5)		
	Total	103 (100)	97 (100)	200 (100)		

Height of keyboard from the floor	Poor	13 (12.6)	18 (18.6)	31 (15.5)	1.344	0.246
	Good	90 (87.4)	79 (81.4)	169 (84.5)		
	Total	103 (100)	97 (100)	200 (200)		

Light intensity of room	Poor	98 (95.1)	74 (76.3)	172 (86.0)	14.754	0.001
	Good	5 (4.9)	23 (23.7)	28 (14.0)		
	Total	103 (100)	97 (100	200 (100)		

Light intensity between participant and computer	Poor	102 (99.0)	73 (75.3)	175 (87.5)	25.809	0.001
	Good	1 (1.0)	24 (24.7)	25 (12.5)		
	Total	103 (100)	97 (100)	200 (100)		

**Table 5:** Comparison between ergonomic practice and CVS.

	Cases	Noncases	Total	*χ*	*p* value
Poor practice	93 (90.3)	66 (68.0)	159 (79.5)	15.175	0.001
Good practice	10 (9.7)	31 (32.0)	41 (20.5)		
Total	103 (100)	97 (100)	200 (100)		

## Data Availability

All data generated or analyzed during this study are included in this article.
